# Genetic architecture of trout from Albania as revealed by mtDNA control region variation

**DOI:** 10.1186/1297-9686-41-22

**Published:** 2009-02-02

**Authors:** Aleš Snoj, Saša Marić, Patrick Berrebi, Alain J Crivelli, Spase Shumka, Simona Sušnik

**Affiliations:** 1 University of Ljubljana, Department of Animal Science, Groblje 3, SI-1230 Domžale, Slovenia; 2 University of Belgrade, Faculty of Biology, Institute of Zoology, Studentski trg 16, 11001 Belgrade, Serbia; 3 Institut des Sciences de l'Evolution, UMR CNRS/UM2 5554, Université Montpellier II, cc065, 34095 Montpellier cedex 05, France; 4 Station biologique de la Tour du Valat, Le Sambuc, 13200 Arles, France; 5 Agriculture University Tirana, Inter faculty Department, Tirana, Albania

## Abstract

To determine the genetic architecture of trout in Albania, 87 individuals were collected from 19 riverine and lacustrine sites in Albania, FYROM and Greece. All individuals were analyzed for sequence variation in the mtDNA control region. Among fourteen haplotypes detected, four previously unpublished haplotypes, bearing a close relationship to haplotypes of the Adriatic and *marmoratus *lineages of *Salmo trutta*, were revealed. Ten previously described haplotypes, characteristic of *S. ohridanus*, *S. letnica *and the Adriatic and Mediterranean lineages of *S. trutta*, were also detected. Haplotypes detected in this study were placed in a well supported branch of *S. ohridanus*, and a cluster of Mediterranean – Adriatic – *marmoratus *haplotypes, which were further delimited into three subdivisions of Mediterranean, *marmoratus*, and a previously non-described formation of four Adriatic haplotypes (Balkan cluster). Haplotypes of the Balkan cluster and the other Adriatic haplotypes, do not represent a contiguous haplotype lineage and appear not to be closely related, indicating independent arrivals into the Adriatic drainage and suggesting successive colonization events. Despite the presence of *marmoratus *haplotypes in Albania, no marbled phenotype was found, confirming previously reported findings that there is no association between this phenotype and *marmoratus *haplotypes.

## Introduction

Major European peninsulas are known to have played a central role in the survival of animal and plants during ice-age maxima and have received a high degree of attention in terms of conservation of endemic taxa [1,2]. Compared with the Iberian and Italian peninsulas, the biodiversity and rich level of phenotypic variability present in the Balkan Peninsula have only recently been investigated by molecular techniques (*e.g. *[3-5]). As one of the 17 biodiversity hotspots of the world [6], this peninsula harbours numerous endemic taxa [4], including members of the genus *Salmo *(subsequently referred to as Balkan trout), which are especially diverse in this region. Many studies on the morphology and phenotypes of the fish of the Balkans were undertaken during the last century (*e.g. *[7,8]) and found high levels of endemism among Balkan trout. As a consequence, given the benefit of availability of modern molecular techniques, a number of recent studies have focused on revisiting Balkan trout taxonomy, population structure and demographic history [9-14]. However, much remains to be done, as the status of several nominal species and populations of Balkan trout remains unresolved, mainly as a result of the region's geographical, political and cultural isolation [15].

Considerable variation in external morphology of Balkan trout was reported in early studies [16,17], giving rise to many taxonomic units (see [18] for review). Recent molecular studies of trout from Bosnia-Herzegovina, Montenegro and FYROM [10,13,14] have confirmed this diversity. However, little clear association between phenotype and genotype has been found, and some well-established taxonomic groups, such as *S. marmoratus *[10], have been found not to be associated with detected genetic assemblages.

Several *Salmo *taxa have been reported to inhabit Albanian rivers and neighbouring drainages in FYROM and Greece. Examples include *S. farioides*, proposed by Karaman [17], and *S. ohridanus*, *S. letnica*, *S. letnica lumi*, *S. trutta*, *S. macrostigma*, *S. peristericus*, *S. marmoratus *and *S. montenegrinus *[19,18,21]. Unfortunately, confirmation of these observations and the continued existence of such trout in these waters, as well as their taxonomic status, remain uncertain, representing an absence from any comprehensive overview of Balkan trout demography, evolution and classification.

The data that do exist on trout in Albania are very scarce and mostly stem from an inventory of fishes undertaken in the country in the 1950s [22], or are restricted to certain areas (*e.g. *[21] on Lake Ohrid; [23] on the River Shkumbini). Rakaj [24] extended and brought up to date the work of Poljakov *et al*. [22] on Albanian ichthyofauna. He described trout from the rivers Shala and Valbona (Ohrid-Drin-Shkodra system; see also [25]) as well as from the lakes Shkodra and Ohrid, while trout have also been reported to exist within the rivers Bistrica [24], Cemit [24], Mati [20] and Shkumbini [23].

Very few genetic analyses of Albanian trout have been performed so far and all are restricted to lakes Ohrid and Prespa [26,13,27].

As inferred from several previous studies on Balkan trout [28,10], anthropogenically induced hybridisation, particularly with introduction of non-native trout lineages, has had a considerable impact on many indigenous trout stocks and has blurred the picture of the original genetic structure and phylogeography of Balkan trout. However, because of Albania's past political isolation and low level of economic development, it is probable that stocking with non-native strains of brown trout (*e.g. *Atlantic lineage) has not been performed here (I Wilson, personal communication). Therefore, despite any impact of over-fishing and intense poaching (authors' personal observations) on the population sizes of native trout, the present distribution and composition of trout in Albania may relatively faithfully reflect the natural situation, a rare situation for salmonid rivers in Europe given the widespread practice of stocking.

In the present survey, we analysed for the first time samples from a both extensive and intensive collection of trout from Albania and from some neighbouring drainages in FYROM and Greece (13 river basins altogether) along with *S. ohridanus *and *S. letnica *from Albanian waters of Lake Ohrid.

The main objective of this study was to determine the genetic architecture of Albanian trout from analysis of the mitochondrial DNA control region (mtDNA CR), and thus obtain phylogeographic information that could be compared with published data and make inferences on the historical demography and evolution of Balkan trout. We also looked for any indication of association between phenotype and mtDNA lineage.

## Methods

### Trout samples collection

In 2005 and 2006, a total of 78 sampling sites were electrofished in rivers in Albania and the Megali Prespa basin in FYROM and Greece. The sampling in Albania (73 sampling sites) was performed not only to undertake a trout census in the country but also for the entire ichthyofauna. Locations were selected based upon observations published in the literature [23,22,20] and from local people. Emphasis was placed on both main water streams and isolated locations.

**Figure 1:**
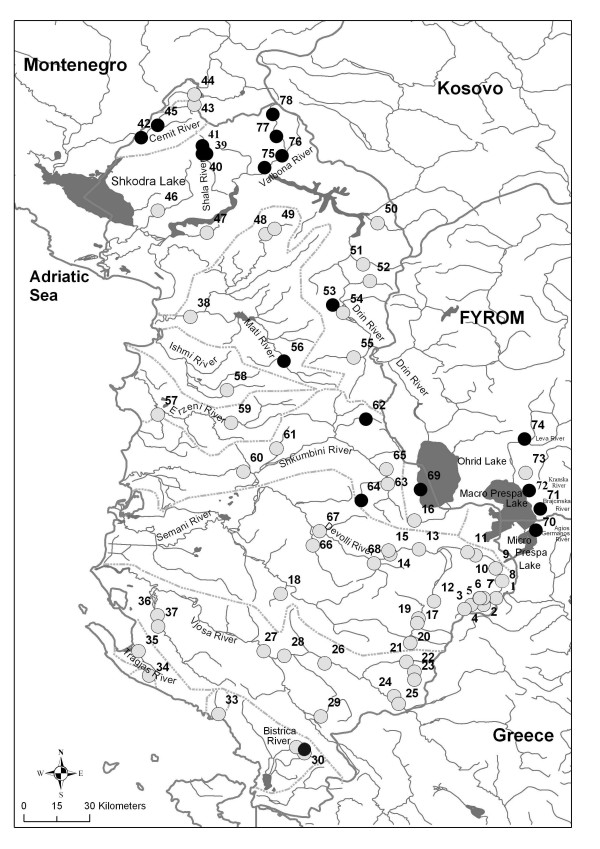
**Map of sample sites**. Sites where trout were found are marked with black (see Table 1); white spots are the sampling stations sampled without trout; dotted lines show the main river catchment.

Among the sampled locations in Albania, trout were found at 15 of them (Fig. 1). Cake and Miho [23] reported trout at sites 61 and 63 (Fig. 1) in the River Shkumbini basin. However, during our sampling campaign, trout were not observed here, though they were found and sampled in two previously non-described locations within this catchment (62 and 64, Table 1). In addition, trout were observed for the first time in the River Mati catchment. On the other hand, they were not found at site 35 (on the River Tragjas), where local people report their existence.

**Table 1:** Sampling locations (numbers as in Figure 1), sample size (N) and haplotype distribution of 14 mtDNA CR haplotypes resolved among 87 trout samples from Albania (AL), Former Yugoslav Republic of Macedonia (FYROM) and Greece

				Haplotype													
Location (see, Figure 1)	Country	N	Taxon	Ad-AL1	Ad-AL2	Ad-AL3	Ma-AL1	Haplo1	Haplo4	Haplo5	Haplo6	ADcs11	Haplo12	Haplo14	AdPrz	MEcs1	ADcs1

L. Ohrid (69)	AL	5	*S. letnica*	-	-	-	-	-	-	-	-	-	5	-	-	-	-

L. Ohrid (69)	AL	5	*S. ohridanus*	-	-	-	-	1	1	2	1	-	-	-	-	-	-

R. Cemit (42, 45)	AL	3	*S. sp.*	-	-	-	-	-	-	-	-	3	-	-	-	-	-

R. Seta (Drin) (53)	AL	8	*S. sp.*	-	-	2	-	-	-	-	-	-	-	6	-	-	-

R. Shala (Drin) (39)	AL	4	*S. sp.*	-	-	-	-	-	-	-	-	3	-	-	1	-	-

R. Zi (Shala, Drin) (40)	AL	3	*S. sp.*	-	-	-	-	-	-	-	-	1	-	-	2	-	-

R. Teth (Shala, Drin) (41)	AL	3	*S. sp.*	-	-	-	-	-	-	-	-	3	-	-	-	-	-

R. Valbona (Drin) (75–78)	AL	11	*S. sp.*	-	3	-	-	-	-	-	-	-	-	1	1	-	6

R. Mati (56)	AL	5	*S. sp.*	-	-	-	-	-	-	-	-	-	-	-	-	5	-

R. Shkumbini (64)	AL	5	*S. sp.*	5	-	-	-	-	-	-	-	-	-	-	-	-	-

R. Qarishta (Skumbini) (62)	AL	6	*S. sp.*	-	-	-	-	-	-	-	-	6	-	-	-	-	-

R. Bistrica (31)	AL	9	*S. sp.*	-	-	-	9	-	-	-	-	-	-	-	-	-	-

R. Brajcino (Prespa) (71)	FYROM	5	*S. peristericus*	-	-	-	-	-	-	-	-	-	-	-	-	-	5

R. Kranska (Prespa) (72)	FYROM	2	*S. peristericus*	-	-	-	-	-	-	-	-	-	-	-	-	-	2

R. Leva (Prespa) (74)	FYROM	5	*S. peristericus*	-	-	-	-	-	-	-	-	-	-	-	-	-	5

R. Agios Germanos (Prespa) (70)	G	8	*S. peristericus*	-	-	-	-	-	-	-	-	-	-	-	-	-	8

Trout were found at four of the five locations sampled in the Megali Prespa basin (in Greece and FYROM), including sites 70 and 71, where, according to the literature [9,29,17], they were expected to exist, and at sites 72 and 74. Trout were not observed at site 73.

The authors' general observation was that poaching for trout was very common in the area sampled, and where trout still exist, the observed densities were very low.

The 87 trout collected from 19 sites (Figure 1 and Table 1) were sorted among three species and one genus: *S. ohridanus *(5), *S. letnica *(5) and *S. peristericus *(20), and *Salmo sp. *(57). While the first two species are easily recognizable on the basis of both their distinct phenotype and specific native range (Lake Ohrid), the classification of *S. peristericus *was based upon its very restricted distribution (see [18]). The other specimens were phenotypically indefinable and were therefore assigned as *Salmo sp*. Mitochondrial DNA haplotypes detected in Albania, Greece and FYROM are reported in Table 1 and all the haplotypes used in phylogenetic analysis are listed in Table 2.

**Table 2:** List of mtDNA CR haplotypes used for phylogeographic analysis and GenBank accession numbers

Haplotype	Acc nb	Haplotype	Acc nb	Haplotype	Acc nb
		
Ad-AL1	EU359770	Ad12	AY653216	MEcs1	AY836350
Ad-AL2	EU359768	ADcs1	AY836330	MEcs10	AY836359
Ad-AL3	EU359769	ADcs11	AY836340	MEcs7	AY836356
AdRc	EU391632	ADcs15	AY836344	ATcs1	DQ841192
AdN	DQ297172	ADcs20	AY836349	AT11a	AY185578
AdPe	DQ318126	Haplo12	AY926570	ATs12	AY836328
AdPrz	DQ318129	Haplo13	AY926573	Das1	AY185568
AdBoz	DQ318128	Haplo14	AY926571	Da2	AY185570
AdTi	DQ318127	Haplo15	AY926572	DaVl	DQ318123
AdC1	DQ381567	Haplo16	DQ381568	Haplo1	AY926564
AdM1	DQ381566	Haplo17	DQ381569	Haplo4	AY926561
AdZ1	DQ381565	Haplo18	DQ381570	Haplo5	AY926569
ADs3	AY260518	Ma-AL1	EU359771	Haplo6	AY926559
Ad4	AY260520	MAcs1	AY836365		
Ad11	AY653218	MA2	AF321995		

### DNA amplification and sequencing

Total DNA was isolated from fin tissue preserved in 96% ethanol following the protocol of Medrano *et al*. [30]. The entire sequence of the mitochondrial DNA control region (mtDNA CR) was amplified by polymerase chain reaction (PCR) using primers 28RIBa [31] and HN20 [32]. Each 30 μL reaction included 1 μM of each primer, 0.2 μM dNTP, 1.5 μM MgCl_2_, 1 × PCR buffer, 1 U *Taq *polymerase (Applied Biosystems) and 100 ng of genomic DNA. The conditions for PCR were initial denaturation (95°C, 3 min) followed by 30 cycles of strand denaturation (94°C, 45 s), primer annealing (52°C, 45 s) and DNA extension (72°C, 2 min). All PCR amplifications were performed in a programmable thermocycler GeneAmp^® ^PCR System 9700 (Applied Biosystems).

Amplified DNA fragments were run on a 1.5% gel and isolated from the gel using the QIAEX II gel Extraction Kit (QIAGEN).

The control region fragment between the tRNA^Pro ^gene and poly T-block of the amplified DNA (100 ng of purified PCR product) was sequenced using primer 28RIBa following ABI PRISM BigDye Terminator protocols (Applied Biosystems 3.1). The amplified DNA was salt-precipitated and analysed with an ABI PRISM 310 automated sequencer.

### Data analysis

Sequences of the 5'-end of the mtDNA CR (ca. 561 bp) were aligned using the computer program ClustalX [33]. To assign individual haplotypes to trout species and lineages previously identified within the brown trout species complex, data were aligned against at least three haplotypes from each lineage (Me: Mediterranean; Ma: *marmoratus*; Da: Danubian; At: Atlantic), and compared to all known haplotypes found in trout samples across the Adriatic river system (Ad; Table 1).

Aligned haplotypes were imported into the program PAUP Version 4.0b10 [34] for phylogenetic analysis. Neighbour-Joining (NJ), maximum parsimony (MP), maximum likelihood (ML) and Bayesian analysis were used for phylogenetic reconstruction. For NJ, a Kimura 2-parameter model was chosen. For MP, insertions or deletions (indels) were included as a fifth character. A heuristic search (10 replicates) with Tree Bisection Reconnection (TBR) branch-swapping was employed to find the most parsimonious trees. For ML, a sequence evolution model was first chosen using the program Modeltest Version 3.7 [35] incorporated into PAUP. After choosing a model, a heuristic search (10 replicates) was used to estimate the most likely topology. Support values for the nodes were obtained with 1000 bootstrap replicates for MP, NJ, or ML analysis, whereby the fast stepwise addition method was used for ML. Bayesian analysis was performed with MrBayes version 3.1.2 [36] where posterior probabilities were obtained using the Markov chain Monte Carlo (MCMC) technique (Nst = 6, Rates = gamma, Ngen = 5,000,000, chains = 4).

Because of weak support for the Adriatic clade as a whole (see Results,) the genealogical relation of these haplotypes was also depicted using a 95% statistical parsimony network constructed from the 5'-end of mtDNA CR sequences using program TCS 1.3 [37]. Resolution of ambiguous loops in the TCS network was performed by comparing ML pair-wise distances of the haplotypes within a loop and identifying the most likely connections within it, reflected by the smallest pair-wise distances. ML pair-wise distances were computed under the model (HKY 85) using the program PAUP Version 4.0b10 [34].

## Results

A total of 561 bp of the mtDNA CR was resolved in 87 individuals and compared with corresponding and already published sequences of various *Salmo *taxa.

In Lake Ohrid, five haplotypes, all previously described in Sušnik *et al*. [38] (marked with "Haplo"), were found, four of which were detected in *S. ohridanus *(Haplo 1, 4, 5 and 6) and one in *S. letnica *(Haplo 12). For the other samples, five already described haplotypes characteristic of the Adriatic (4) and Mediterranean (1) lineages of *S. trutta *were found. In addition, four previously unpublished haplotypes bearing close relation to others of the Adriatic (Ad-AL1 to 3) and *marmoratus *(Ma-AL1) were also detected.

*Salmo peristericus *from the FYROM part of the Prespa basin were fixed for haplotype ADcs1; this haplotype was also found in the River Valbona system in Albania (River Drin basin). Haplotype AdPrz was found in the rivers Valbona and Shala (also River Drin basin).

Haplo14, previously considered private for *S. letnica *in Lake Ohrid [13], was in this study found to exist also in trout in the Drin basin.

It is worth noting that all the samples from the River Bistrica were fixed for Ma-AL1, but none exhibited any phenotypic character state characteristic of *S. marmoratus *(field observations).

The highest level of genetic variation appeared to be in the Drin basin (haplotypes ADcs1, AdPrz and Haplo14) and the most common haplotype found in this study was ADcs1, found in Lake Prespa tributaries and the River Valbona.

**Figure 2:**
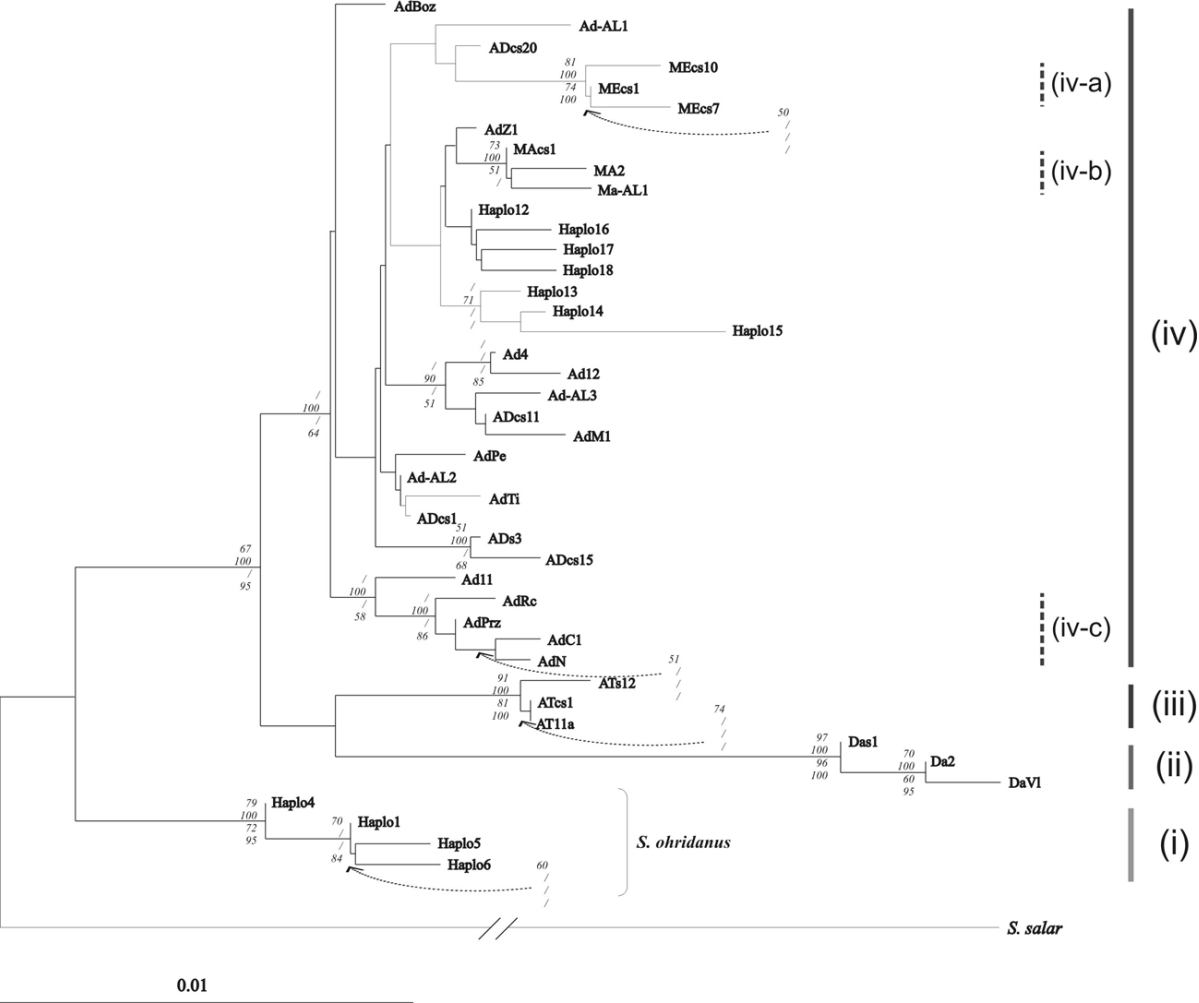
**Neighbour-joining (NJ) tree for genus *Salmo *based on 561 bp of the 5'-end mtDNA control region and on a Kimura 2-parameter substitution model**. In addition to haplotypes characteristic of Balkan trout from the Adriatic and Aegean drainages (Ad), three haplotypes representing Mediterranean (Me), Danubian (Da) and Atlantic (At) drainages were included in the analyses; haplotypes characteristic for Lake Ohrid *S. letnica *are marked with "Haplo"; bootstrap support values refer, from top to bottom, to NJ, maximum parsimony, maximum likelihood (HKY+I+G model, transition: transversion 2.6389; proportion of invariable sites (I) 0.6060; gamma distribution shape parameter 0.7375) and Bayesian methods; values <50 are marked with "/" or are not marked when there was no value above 50 in any of the analyses.

The phylogenetic organisation of the NJ distance tree clearly identified four well supported branches (Fig. 2): (i) *S. ohridanus *with Haplo1, 4, 5 and 6, (ii) the reference Danubian haplotypes, (iii) the reference Atlantic haplotypes, and (iv) a cluster of Mediterranean, Adriatic and *marmoratus *(ME-AD-MA) haplotypes exhibiting a very complex but poorly supported clade. All previously unreported haplotypes appeared in this clade.

Character state phylogenetic (*i.e.*, MP, ML and Bayesian) analyses revealed similar tree-topology with regard to the four main clades (Fig. 2) and provided a better resolution of the ME-AD-MA clade showing clear delimitation of three subdivisions: two already accepted groups of Mediterranean (iv-a) and *marmoratus *(iv-b) haplotypes [39,40], and (iv-c), a previously non-described formation of haplotypes AdRc, AdPrz, AdC1 and AdN (hereafter referred to as the Balkan cluster). The topology of the other Adriatic haplotypes remained largely unresolved.

**Figure 3:**
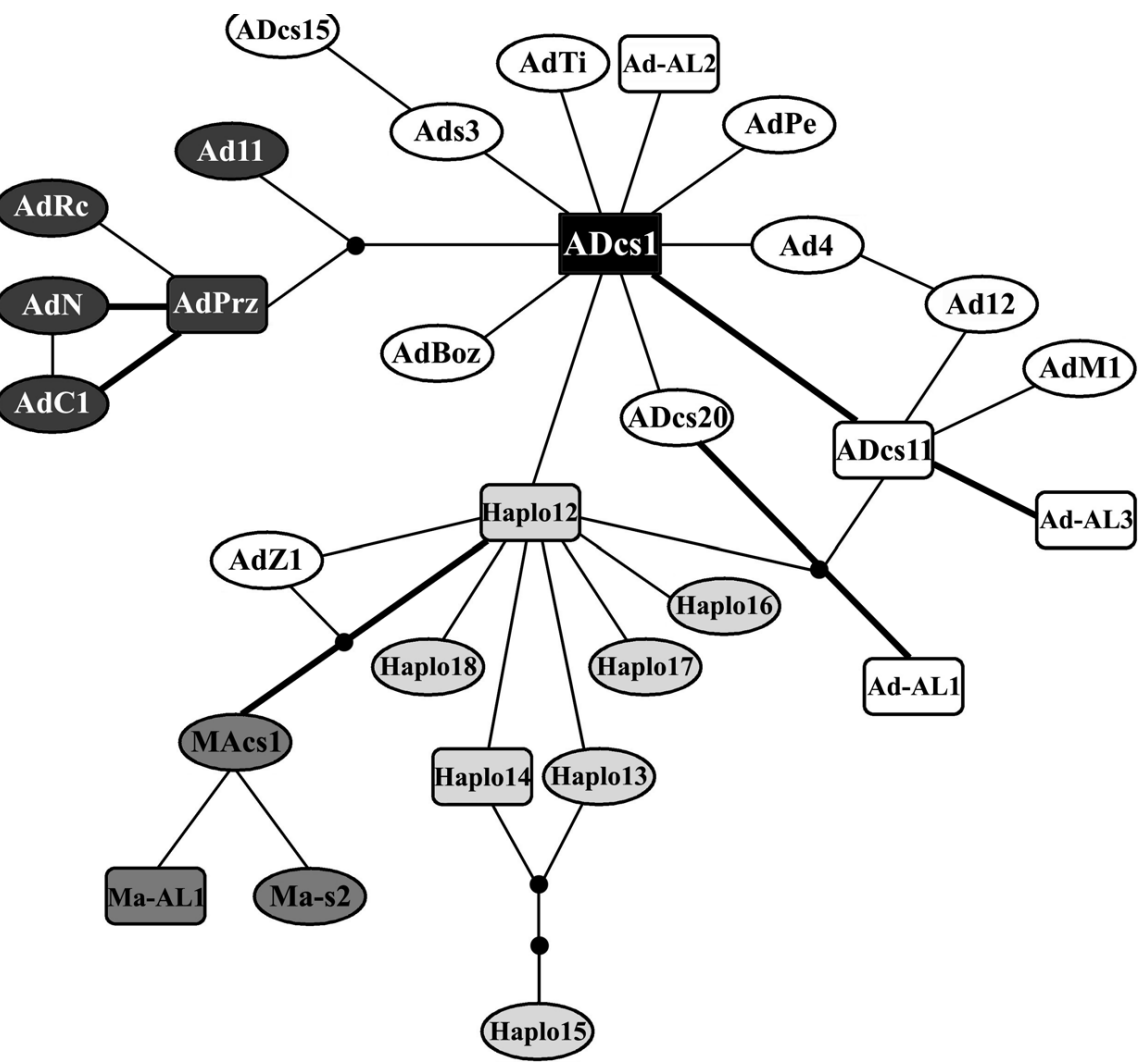
**Haplotype network relating the Adriatic clade haplotypes found in Albanian trout populations (561 bp of the CR 5'-end) with previously published data **[40,13]. Lines, regardless of length, represent single mutational events and link the haplotypes; black dots represent missing or theoretical haplotype; haplotypes found in Albania are in a square, those from Lake Ohrid are in light grey, those linked to *S. farioides *in black, while Ma haplotypes are in dark grey; most likely connections of the haplotypes within ambiguous loops, based on ML pair-wise distances are displayed with thick lines

A network gathering the haplotypes found in this study and those previously published [40,13] is presented in Figure 3. Haplotype MEcs1 was found to exhibit several autapomorphies, which separated it considerably from other haplotypes and complicated the resolution of the network (data not shown). For this reason, this haplotype was excluded from further analysis. The general organization of the haplotype network obtained in this study featuring a multiple star-like structure with ADcs1 taking a central position was similar to the one reported by Cortey *et al*. [40] and confirmed in Sušnik *et al*. [13]. Those newly described are only one (Ad-AL2, Ad-AL3 and Ma-AL1) or two (Ad-AL1) mutation steps away from previously described haplotypes. AdPrz, a common haplotype in the southern Adriatic drainage [41], and AdRc and AdC1 from Lake Shkodra basin [13] and AdN from the River Neretva basin [10] form a separate group in the network, supporting the existence of the corresponding clade inferred from the phylogenetic tree (iv-c, Fig. 2). Interestingly, *marmoratus *haplotypes were found to be incorporated into the Adriatic clade network being apparently closely related to haplotypes predominantly detected in Ohrid trout (*S. letnica*).

## Discussion

This study reveals for the first time the phylogenetic structure of trout populations in one of the last remaining incompletely explored regions of trout distribution in Europe. Drainages in Albania are linked to neighbouring systems in FYROM and Greece that also belong to the Adriatic river system as a whole. These rivers and lakes have been largely unmanaged with respect to stocking of non-native strains of trout, in contrast with most of the rest of Europe. However, stocking with trout from the same location has been practised over many years in Lake Ohrid, and in at least one tributary of Lake Prespa. Therefore, it was expected that non-native genetic signatures would not be detected and, indeed, this was the case: *e.g. *no haplotypes of Atlantic or Danubian brown trout phylogenetic lineages were found. Instead, Albanian trout populations are characterised by mtDNA haplotypes from the three other previously defined brown trout lineages [39]: Adriatic, Mediterranean and *marmoratus*. All of these three lineages are native to Mediterranean river systems. Moreover, Lake Ohrid contains the endemic species *S. ohridanus *(more closely related to *S. obtusirostris *of the Dalmatian river systems), with its unique haplotypes. Such pronounced genetic diversity places Albanian trout populations among the most variable in Europe. This finding is even more remarkable when one considers the very limited geographic distribution of these trout in Albania and the neighbouring area. Out of 78 locations sampled, we expected to find trout in at least 25 of them but were able to catch trout only in 19 sites. With few exceptions (sites 39,40,41) trout were always at very low density and it was difficult to catch more than five in several stations, probably as a result of heavy poaching by net, dynamite and chlorine. Consequently, although trout also exist in remote areas they are in danger of extinction.

### Congruence between taxonomic group and mtDNA lineage

Lake Ohrid, the oldest lake in Europe, is shared between FYROM and Albania. According to historical data [21] and recent studies [11,38,13,27] at least two trout species, *S. ohridanus *and *S. letnica*, inhabit the lake and samples of both were included in this survey. Genetic analysis, including of mtDNA, has already been performed for these two taxa [38,13]. The results obtained in the present study corroborate the earlier findings, justifying the distinct taxonomic position of *S. ohridanus *in relation to its congeners, and supporting the species status of Ohrid trout *S. letnica *for conservation purposes [13]. The present study found a high frequency of Haplo14 in the Drin river system (previously reported only for Lake Ohrid), an unsurprising finding since until 1960 no dams existed along this river and Lake Ohrid was connected directly with rest of the system.

The second largest lake in the region, of Tertiary origin (>5 MY), is Lake Megali Prespa, whose tributaries are inhabited by *S. peristericus *[17]. During the Jurassic, lakes Prespa and Ohrid formed part of the Dassaretic lakes, which were linked with the Adriatic Sea. Trout from the River Agios Germanos, now a tributary of this lake after the stream was diverted from Lake Mikri Prespa between 1935 and 1945, have already been analysed at allozyme loci [42] and for mtDNA sequence variation [26]. On the basis of a diagnostic allele at CK-2*, this population was first thought to be distinct from other populations of brown trout in Greece. However, based on subsequent partial mtDNA CR and cyt b gene sequence analysis, it was then placed back in the *S. trutta *complex. All the populations of *S. peristericus *examined in the present study were fixed for the haplotype ADcs1, which distinguished them from all trout populations surveyed here apart from the population from the River Valbona (Drin basin), where this haplotype was also found. A recent hypothesis concerning demographic patterns of the Adriatic lineage [40,14] considers ADcs1 as the central haplotype (Fig. 3): it is the most common haplotype in the Iberian Peninsula, where the Adriatic lineage is thought to have originated [40]. The plesiomorphic state of the haplotype ADcs1 and its presence in *S. peristericus *indicates its ancestry within the Adriatic lineage and does not support the recognition of this taxon as a separate species. Nevertheless, given that *S*. *peristericus *is distinct morphologically from all other Balkan trout [9] and restricted geographically to Lake Megali Prespa basin, we stress the importance of this taxon as a unit that needs conserving.

No marbling phenotype characteristic of marble trout was observed in any of the individual trout caught in the present study, even though *S. marmoratus *has been reported to be present in the rivers Valbona and Drin [19,24,25]. On the other hand, the *marmoratus *haplotype (Ma-A1) was detected in southern Albania, in the River Bistrica where Rakaj [20] has described the local form as *S. peristericus*. As no marbling was observed in the trout from this river, this supports the view that the *marmoratu*s mtDNA lineage and the marbling phenotype are not linked: previous reports have described the existence of *marmoratus *haplotypes in many populations of phenotypically brown trout across the Mediterranean river basins, including rivers in Dalmatia [39], central Italy [43], Greece [26] and Corsica (unpublished data). The marbling phenotype is only characteristic of this lineage in its north Adriatic range, where the phenotype was first described [44,45].

### Phylogeographic considerations

Much effort has been put in resolving salmonid genetic structure and phylogeographic signals in the Adriatic drainage [41,10,43,38,14]. To update the overall current picture, genetic data on samples from Albania and neighbouring freshwater systems in FYROM and Greece can now be incorporated into previously obtained data and used to make inferences on trout phylogeography and historical demography in the western Balkans and supplement the current knowledge concerning the rest of the Adriatic drainage.

The presence of four evolutionary lineage haplotypes (of *S. ohridanus*, *S. marmoratus*, and Adriatic and Mediterranean *S. trutta*) in Albania points to a complicated demographic history and rich diversity of trout populations in the country. The lineages AD and ME, both reported here as present in Albania, have been studied and reviewed thoroughly by Cortey *et al*. [40] who suggested that they had originated in the Iberian peninsula some 150,000 years ago, with haplotypes ADcs1 and MEcs1 as the most ancestral, respectively. It is thought that these lineages expanded together from west to east across the Mediterranean basin during the extreme Pleistocene glacial maxima and therefore would have reached the western Balkans relatively recently. In Albania, the ME haplotype (MEcs1) was found in a single river basin (River Mati), and was the only haplotype present there. Indications of a patchwork distribution of ME haplotypes have already been reported for the western Mediterranean drainages [40], the Aegean and Adriatic drainages in Greece [26] and in central Italy [43]. As Albanian rivers represent the limit of the geographical range of the ME lineage, it seemed likely that here the concentration of such haplotypes would be low and that they would be very sensitive to stochastic events (gene flow, bottlenecks, etc.). Such events appear to have been particularly intense in the Balkan Peninsula during the Pleistocene [4] and could be the main reason for both the present geographical limitations of distribution and local fixation of the MEcs1 haplotype.

A complex and particularly fuzzy phylogenetic relationship among AD haplotypes, already observed by Cortey *et al*. [40] and Sušnik *et al*. [13], was noticed in this study, and only the so-called Balkan cluster (haplotypes AdN, AdRc, AdC1 and AdPrz) was well resolved, with a bootstrap value of 100 per cent in MP and 86 per cent in Bayesian. This cluster corresponds to the AdN-AdPrz cluster previously described by Marić *et al*. [41] and Razpet *et al*. [10] for the rivers Neretva and Prizrenska Bistrica. In those studies, the distribution of haplotypes corresponded well with the distribution of the questionable taxon *S. farioides *(from rivers Krka (Croatia), Neretva (Bosnia and Herzegovina) and Prizrenska Bistrica (Kosova), tributaries of Lake Shkodra (Montenegro) and of the River Drin and Lake Ohrid) [17,9]. Two additional haplotypes constituting the Balkan cluster and used here as reference haplotypes, AdRc and AdC1, also originate from the *S. farioides *range (Lake Shkodra tributaries; see [13]) and additionally support the proposed haplotype-species association. In this study, the haplotype AdPrz was found in the River Drin basin, the eastern limit of the range of *S. farioides*, and close to the type location of Prizren, which shares the same water shed. Thus, the results from this study support the congruence of the distribution of the Balkan haplotype cluster and the range of *S. farioides*. As reported previously [10,13], haplotypes of the Balkan cluster, including AdPrz, on the one hand and other Ad haplotypes on the other hand, do not represent a contiguous haplotype lineage (see Fig. 3) and appear not to be closely related, indicating independent arrivals into the Adriatic drainage and suggesting successive colonization events.

The data referring to the distribution of the *marmoratus *haplotype in Albania do not contribute much to resolving contradictory notions about the centre of origin and demographic patterns of *marmoratus *lineage (c.f., [46,39,48]). The newly described Ma-AL1 haplotype, recorded for a previously non-surveyed location in Albania, broadens the known genetic diversity of the *marmoratus *lineage, and highlights its extensive but patchy distribution, as observed across a broad stretch of Mediterranean river systems [26,39,43].

Balkan trout are composed of a genetic mosaic of haplotypes, related to most of the other trout lineages of the Mediterranean area analysed and reported in other studies. However, due to a complexity of past migrations, colonisations and extinctions, as well as that of many other organisms [4], the Balkans has been considered a hotspot of trout biodiversity. The region's unique mix of habitats and topography has created a peninsula rich in endemism, and ironically its isolation (both physical and political) has helped to conserve a complex structure of trout populations, particularly in Albania. This first investigation of a little explored area has revealed a glimpse into a partly understandable and partly fuzzy web of relationships.

## Competing interests

The authors declare that they have no competing interests.

## Authors' contributions

AS participated in the study design and coordination and drafted the manuscript. SM carried out the molecular genetic studies and prepared the sequence alignment. PB participated in the design and coordination of the study and in writing the manuscript. AJC conceived the study, succeeded in finding funding, participated in its design and coordination, and helped to draft the manuscript. SSh organized the logistic for the fieldwork, participated with the collection of data and helped to draft the manuscript. SS carried out phylogenetic analyses and helped to draft the manuscript. All authors read and approved the final manuscript.
